# Defecation status, intestinal microbiota, and habitual diet are associated with the fecal bile acid composition: a cross-sectional study in community-dwelling young participants

**DOI:** 10.1007/s00394-023-03126-8

**Published:** 2023-03-07

**Authors:** Yosuke Saito, Toyoaki Sagae

**Affiliations:** 1 Present Address: Department of Clinical Nutrition, Faculty of Health and Wellness Sciences, Hiroshima International University, 5-1-1, Hirokoshingai, Kure, Hiroshima 737-0112 Japan; 2 Department of Human Life Sciences, Sakura no Seibo Junior College, Fukushima, Japan; 3 Department of Health and Nutrition, Yamagata Prefectural Yonezawa University of Nutrition Sciences, Yamagata, Japan

**Keywords:** Fecal bile acid, Intestinal microbiota, Defecation frequency, Animal fat, Insoluble fiber, *Clostridium* cluster XIVa

## Abstract

**Purpose:**

Bile acid (BA) metabolism by intestinal bacteria is associated with the risk of gastrointestinal diseases; additionally, its control has become a modern strategy for treating metabolic diseases. This cross-sectional study investigated the influence of defecation status, intestinal microbiota, and habitual diet on fecal BA composition in 67 community-dwelling young participants.

**Methods:**

Feces were collected for intestinal microbiota and BA analyses; data about defecation status and dietary habits were collected using the Bristol stool form scales and a brief-type self-administered diet history questionnaire, respectively. The participants were categorized into four clusters based on their fecal BA composition, according to cluster analysis, and tertiles based on deoxycholic acid (DCA) and lithocholic acid (LCA) levels.

**Results:**

The high primary BA (priBA) cluster with high fecal cholic acid (CA) and chenodeoxycholic acid (CDCA) levels had the highest frequency of normal feces, whereas the second BA (secBA) cluster with high levels of fecal DCA and LCA had the lowest. Alternately, the high-priBA cluster had a distinct intestinal microbiota, with higher *Clostridium* subcluster XIVa and lower *Clostridium* cluster IV and *Bacteroides*. The low-secBA cluster with low fecal DCA and LCA levels had the lowest animal fat intake. Nevertheless, the insoluble fiber intake of the high-priBA cluster was significantly higher than that of the high-secBA cluster.

**Conclusion:**

High fecal CA and CDCA levels were associated with distinct intestinal microbiota. Conversely, high levels of cytotoxic DCA and LCA were associated with increased animal fat intake and decreased frequency of normal feces and insoluble fiber intake.

**Clinical trial registry:**

University Hospital Medical Information Network (UMIN) Center system (UMIN000045639); date of registration: 15/11/2019.

**Supplementary Information:**

The online version contains supplementary material available at 10.1007/s00394-023-03126-8.

## Introduction

Primary bile acids (priBAs)—including cholic (CA) and chenodeoxycholic acids (CDCA)—are synthesized from cholesterol in the human liver, conjugated with either taurine or glycine, and secreted into the intestinal tract, where they dissolve dietary lipids [1]. Conjugated priBAs are then reabsorbed in the terminal ileum and returned to the liver [2]. Some conjugated priBAs are modified by intestinal bacteria in a stepwise manner [3]. Starting with a deconjugation of priBAs to free priBAs by intestinal bacteria with the aid of bile salt hydrolase activity [4], the free priBAs are then converted to various secondary BAs (secBAs) [2]. Intestinal BA metabolism is associated with the incidence and progression of various diseases [5, 6]. Thus, modifying the intestinal BA metabolism could emerge as a new strategy for preventing or treating several diseases [7].

Intestinal bacteria converted CA and CDCA via 7α-dehydroxylation into deoxycholic acid (DCA) and lithocholic acid (LCA), respectively, which are the predominant BAs in human large bowel [2]. DCA and LCA are potentially genotoxic and tumor-promoting [8]. Their increased production has been associated with a similarly increased risk of colon cancer [9], cholelithiasis [10], and liver cancer [11]. Alternatively, as BAs help regulate lipid and glucose metabolism via the farnesoid X receptor, use of antibiotics to inhibit secBA production reduced serum triglyceride and glucose levels [12]. Probiotics may also help lower serum cholesterol levels [13, 14] due to increased fecal excretion. This is accomplished through increased free priBA production and low reabsorption in the terminal ileum [15]. However, this theory is controversial because free priBAs might be further converted to toxic secBAs within the colon [16]. Additionally, increased exposure to BAs in the colon might be associated with diarrhea by enhancing the secretion of fluids and electrolytes [17]. On the other hand, low levels of *Bacteroidetes*, bacteria that predominantly produce bile salt hydrolase [18], have been found in patients with obesity [19]. Additionally, low levels of *Clostridium* subcluster XIVa, to which bacteria with 7α-dehydroxylating activity belong, are associated with intestinal dysbiosis [20]. Although BAs are modified by intestinal bacteria, it remains unclear how the relative abundance of various strains of bacteria affect human health [21].

Diet is a modifiable factor that can influence defecation status [22], BAs [23], and intestinal microbiota [24, 25]. Defecation status, BAs, and intestinal microbiota may interact [17, 20, 26, 27]. Given known associations between various BAs and disease prevention or treatment, we sought to clarify the relationships that exist among diet, defecation status, BAs, and intestinal microbiota. Unfortunately, few studies have examined all these factors. Additionally, BA studies conducted with animal models are limited by known differences between humans and non-human animals [28]. Specifically, the main priBAs in rats are CA and β-muricholic acid; whereas, in humans the main priBAs are CA and CDCA. Thus, human studies may be more relevant. This cross-sectional study identified associations among defecation status, intestinal microbiota, and diet by examining fecal BA composition in community-dwelling young participants.

## Methods

### Study participants

The study participants were 70 students enrolled at the Yamagata Prefectural Yonezawa University of Nutrition Sciences or Sakura no Seibo Junior College. Two participants who had taken medications for diarrhea or constipation during the week before fecal sampling were excluded from the analysis. Additionally, during cluster analysis, we excluded a third participant who did not fit any cluster. Therefore, 67 participants were included in the analysis (5 males, 62 females; age: 18–22). All participants provided written informed consent; the study protocol was approved by the Ethics Committee of the Yamagata Prefectural Yonezawa University of Nutrition Sciences (Approval No. 2019–9), and the study protocol was according to the Declaration of Helsinki.

### Protocol

The participants underwent the following examinations one time each: (A) recording of defecation status and probiotic foods consumed the week before the fecal collection, (B) fecal collection, (C) dietary assessment using a brief, self-administered diet history questionnaire (BDHQ) [29, 30]. The participants were instructed to carry on with their typical routines while in the study and not make any major changes to their general diet or physical activity.

#### Records of defecation status and probiotic foods

The participants recorded their defecation status using the Bristol stool form scale (BSFS) for 1 week before fecal collection. BSFS is a tool designed to classify fecal form into seven categories (types 1–2 for hard feces, types 3–5 for normal feces, and types 6–7 for watery feces). This tool is widely used in clinical and research fields [31, 32]. The participants were instructed about the BSFS and asked to self-assess and record the form of their feces immediately after defecation. They were asked to also record the probiotic foods (defined as foods containing lactic acid bacteria and bifidobacteria) taken for this period. These records were kept until the feces were collected.

#### Collection and analysis of feces

We asked the participants to collect their feces at a convenient time after a week of recording their bowel movements and intake of probiotic foods. Following the instructions, the participants collected all defecated feces immediately in a special fecal collection cup, stirred them several times with a spoon, and then placed the collection cup in a container, which in turn, was set in a cool bag with the coolant. The participant also evaluated their collected feces using the BSFS. The participants submitted the bag to the researcher as soon as possible (i.e., samples collected in the early morning on weekdays were submitted in the morning; samples collected on campus were submitted promptly; if the samples were collected on a holiday or at night, the participant contacted the researcher, who then picked up the sample at the participant’s home). The submitted fecal samples were stored at − 80 °C until use.

We analyzed fecal microbiota by Techno Suruga Laboratory Co., Ltd. (Shizuoka, Japan), using terminal restriction fragment length polymorphism (T-RFLP) targeting the bacterial 16S rDNA. DNA were extracted from the fecal samples according to a previously published protocol [33]. Briefly, 100 mg of each fecal sample was suspended in 4 M guanidine thiocyanate, 100 mM Tris–HCl (pH 9.0), and 40 mM EDTA and then beaten with zirconia beads using a FastPrep-24 5G instrument (MP Biomedicals, USA) to obtain crude extracted DNA. The DNA was purified using an automated DNA isolation system (GENE PREP STAR PI-480, Kurabo Industries, Japan), and a DNA isolation reagent kit (NR-201, Kurabo Industries, Japan). We estimated DNA concentrations using the NanoDrop ND8000 (Thermo Ficher Scientific, USA) and adjusted the final DNA sample concentration to 10 ng/μL. We performed amplification of 16S rDNA, restriction enzyme digestion, and fragment analysis according to a previously published protocol [34, 35]. Here 16S rDNA was amplified using a fluorescent-labeled 516f primer (5′-TGCCAGCAGCCGCGGTA-3′) and 1510r primer (5′-GGTTACCTTGTTACGACTT-3′) The resulting 16S rDNA amplicons were digested with FastDigest BseLI (BslI, Thermo Fisher Scientific, USA) for 10 min. The digested products were subjected to fragment analysis via the ABI PRISM 3130xl Genetic Analyzer System (Applied Biosystems, USA). The taxonomy of the clostridial species was classified into clusters, as proposed by Collins et al. [36, 37].

We analyzed fecal BA concentrations by Techno Suruga Laboratory Co., Ltd. (Shizuoka, Japan) using liquid chromatography in combination with hybrid quadrupole time-of-flight mass spectrometry (LC-QTOF-MS). BAs were extracted from fecal samples using a method previously described [38] with minor modifications; 100 mg of each fecal sample were suspended in 0.9 mL of sodium acetate buffer (100 mM, pH 5.6) mixed with ethanol using a 2 mL tube with zirconia beads and then heat-treated at 85℃ for 30 min. After centrifugation at 18,400×*g* for 10 min, the supernatant was diluted fourfold with water and applied to the solid-phase extraction using a Bond Elut C18 cartridge (Agilent Technologies, USA). The solvent of the obtained extract was evaporated, and the residue was dissolved in 50% ethanol with internal standard. This solution was filtered through a hydrophilic polytetrafluoroethylene filter and used as a sample for LC-QTOF-MS analysis. The LC-QTOF-MS instrument comprises Waters ACQUITY UPLC, Xevo G2-S QTOF, and an electrospray ionization probe (waters, USA). An Acquity UPLC BEH C18 column (1.7 µm, 2.1 × 150 mm, Waters, USA) was used at 65℃. Gradient elution performed the separation using 0.1% formic acid aqueous solution (solvent A) and acetonitrile containing 0.1% formic acid (solvent B) at a flow rate of 0.5 mL/min. The gradient elution program for solvent B is as follows: 0–0.5 min, 30%; 0.5–1.0 min, 30–35%; 1.0–7.0 min, 35–40%; 7.0–10.0 min, 40–50%; 10.0–11.5 min, 50–95%; and 11.5–13.0 min, 95%. The QTOF mass spectrometer operated in negative ion mode. The desolvation gas was nitrogen, the collision gas was argon, and the following parameters were used: capillary voltage, 0.5 kV; sampling cone voltage, 20 V; source temperature, 150 ℃; desolvation temperature, 450 ℃; cone gas flow, 100 L/h; desolvation gas flow, 1000 L/h; scan time, 0.3 s; and data acquisition region, 50–850 m/z. Leucine enkephalin was used as lock mass, which generated a 554.2615 Da [M-H]- ion.

#### Assessment of habitual diet

We assessed participants’ diets during the preceding month using the BDHQ [29, 30]. This questionnaire, based primarily on the Standard Table of Food Composition in Japan and formulated by Japan MEXT [39], asks how frequently the respondent consumes 58 different foods and beverages. A commercial computer algorithm was used to calculate nutritional intake. Because the BDHQ is able to rank the energy-adjusted intake of many nutrients [29, 30], each participant’s consumption of various food items was expressed as density per 1000 kcal.

### Categorization based on the fecal BAs composition

We categorized the participants of the study based on their fecal BAs composition by combining cluster analyses and tertiles. The 17 types of BA concentrations we analyzed were subjected to subsequent cluster analysis [including five free BAs (CA, CDCA, DCA, LCA, and ursodeoxycholic acid (UDCA)), five glycine conjugated (G-) BAs (G-CA, G-CDCA, G-DCA, G-LCA, and G-UDCA), five taurine conjugated (T-) BAs (T-CA, T-CDCA, T-DCA, T-LCA, and T-UDCA), 7-oxo-DCA, and 7-oxo-LCA]. The data matrix is presented as Online Resource 1. We used non-standardized variables because they were all on the same scale (µmol/g), and the data were comparable. First, we determined the number of clusters through the tree diagram, which is generated using the squared Euclidean distance via the ward’s method [40]. Thereafter, participants were categorized using K-means cluster analysis based on squared Euclidean distances. The K-means method is one of the most widely used clustering methods and requires the number of clusters to be set in advance [41]. Subsequently, one obtained cluster was further divided into tertiles based on the total concentrations of DCA and LCA.

The fecal bile acid levels were measured per fresh fecal mass and were therefore affected by the fecal water content. Thus, we analyzed the categorization scheme generated by the cluster analysis, which also included the BSFS type of feces used in the analysis as a variable. Two-step cluster analysis, which can also handle categorical variables, was used. All categories obtained by cluster analysis including the BSFS type were identical, with the exception of one participant’s classification (see Online Resource 2), and the results of subsequent analyses were similar. All cluster analyses were performed using the Statistical Package for the Social Sciences Software Ver. 28.0 for Windows (IBM SPSS 28 Statistics Base, Inc., Chicago, IL, USA).

### Statistical analysis

Characteristics of the study participants were presented as mean ± standard deviation (SD) or percentage. Between-cluster differences were assessed using the one-way analysis of variance (ANOVA). We used Tukey’s test for post hoc pairwise multiple comparisons if Levene's test showed homogeneity of variance; the Games–Howell’s test was used for samples with non-homogeneous variances. The data are presented as mean ± SD. To better interpret the association of defecation status, intestinal microbiota, and habitual diet with fecal BA composition, we performed principal component analysis (PCA) on some variables that showed significant differences among clusters based on the fecal BAs composition. The variables used in the PCA were shown in Online Resources 3. All statistical analyses were performed using the Statistical Package for the Social Sciences Software Ver. 28.0 for Windows (IBM SPSS, Inc., Chicago, IL, USA); *p* values of < 0.05 are considered indicative of statistical significance.

## Results

### Characteristics of the participants

**Fig. 1:**
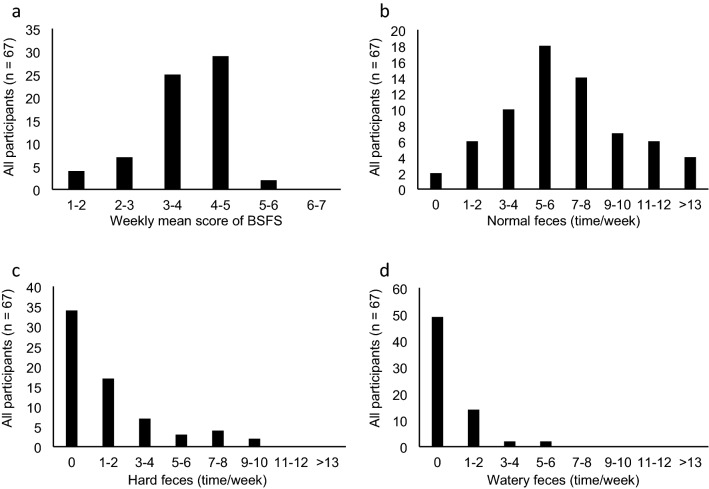
Defecation status for a week in study young participants living in the community. The participants assessed and recorded all feces excreted in the week using the BSFS. **a** Distribution of weekly mean score of BSFS for each participant. **b** Distribution of frequency of excretion of normal feces (BSFS type 3–5). **c** Distribution of frequency of excretion of hard feces (BSFS type 1–2). **d** Distribution of frequency of excretion of watery feces (BSFS type 6–7). *BSFS* Bristol stool form scale

The characteristics of the participants are shown in Online Resource 4. The mean age was 19.9 years, with a disproportionate number of females (92.5%). Their mean BMI was 21.2 kg/m^2^, and 77.6% were within normal limits for body weight (18.5 ≤ BMI < 25). Participants’ defecation status is presented as Fig. 1; 80.6% of the participants had a mean BSFS score of 3 ~ 5 (normal feces) for one week (Fig. 1a). The mean ± SD of the frequency of normal feces was 6.7 ± 3.9 time/week (Fig. 1b); 50.7% and 73.1% of the participants had no hard and watery stools during the week, respectively (Fig. 1c, d).

**Fig. 2:**
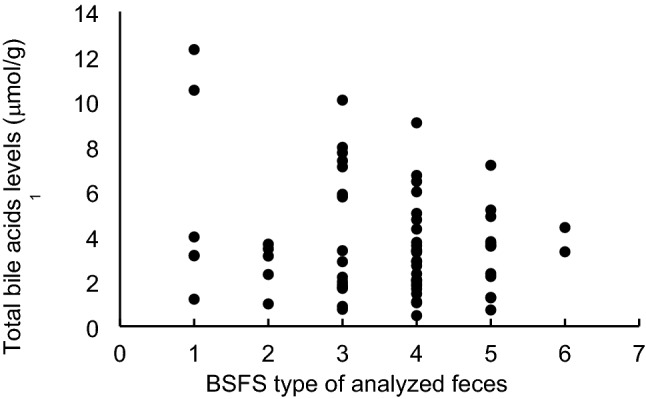
Distribution of total bile acid levels according to the form of the analyzed feces. The form of the feces used in the analysis was assessed and recorded by the participant using the BSFS at the time of fecal collection. BSFS types 1 and 2 indicate hard stools, 3–5 indicate normal stools, and 6 and 7 indicate watery stools. *BSFS* Bristol stool form scale. ^1^Bile acid levels were measured per fresh fecal mass

The distribution of total BA levels, according to fecal form, is shown in Fig. 2. The feces of 80.6% (n = 54) were considered normal, 16.4% (n = 11) hard, and 3.0% (n = 2) watery. There were no significant differences in fecal total bile acid levels relative to BSFS type (ANOVA, *p* = 0.499, data not shown).

Table 1 shows the participants’ fecal characteristics. Out of 17 BAs measured, DCA and LCA were predominant, with mean values of 1.57 and 0.89 µmol/g, respectively; 7-oxo-LCA, five G-BAs, and five T-BAs were detected only in the feces of two, four, and six participants, respectively. The *Bacteroides*, *Clostridium* cluster XIVa, and *Bifidobacterium* were predominant, with mean relative abundance values of 29.9%, 22.8%, and 19.4%, respectively.

**Table 1:** Bile acid levels and microbiota in the feces of study participants living in the community

All participants (n = 67)	
Bile acid levels (µmol/g)^a^	
Total bile acids	3.73 ± 2.55
CA	0.56 ± 0.95
CDCA	0.34 ± 0.71
DCA	1.57 ± 1.44
LCD	0.89 ± 0.95
UDCA	0.21 ± 0.36
7-oxo-DCA	0.10 ± 0.21
7-oxo-LCA	0.02 ± 0.13
Glycine conjugated bile acid^b^	0.03 ± 0.22
Taurine conjugated bile acid^b^	0.01 ± 0.03
Microbiota (%)	
*Bifidobacterium*	19.4 ± 12.4
*Lactobacillales* (Order)	7.4 ± 6.1
*Bacteroides*	29.9 ± 13.1
*Prevotella*	0.2 ± 1.5
*Clostridium* cluster IV	6.7 ± 4.2
*Clostridium* subcluster XIVa	22.8 ± 11.1
*Clostridium* cluster IX	3.9 ± 5.1
*Clostridium* cluster XI	0.5 ± 1.0
*Clostridium* cluster XVIII	1.2 ± 2.4
Others	8.0 ± 5.0
Firmicutes/Bacteroidetes ratio	2.4 ± 3.3

Data presented as mean ± standard deviation. Data are referred to a single sample per participant

*CA* cholic acid, *CDCA* chenodeoxycholic acid, *DCA* deoxycholic acid, *LCA* lithocholic acid, *UDCA* ursodeoxycholic acid

^a^Bile acid levels were measured per fresh fecal mass

^b^Glycine and taurine conjugated bile acid each contained five bile acids (CA, CDCA, DCA, LCA, and UDCA)

### Categorization of the participants

**Fig. 3:**
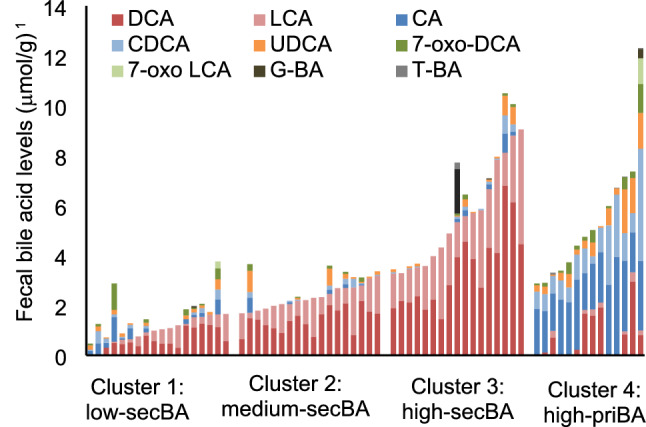
Profiling of fecal bile acids of the participants living in the community. *DCA* deoxycholic acid, *LCA* lithocholic acid, *CA* cholic acid, *CDCA* chenodeoxycholic acid, *UDCA* ursodeoxycholic acid, *G-BA* glycine conjugated bile acid, *T-BA* taurine conjugated bile acid, *priBA* primary bile acid, *secBA* secondary bile acid. G-BA and T-BA each contained five bile acids (CA, CDCA, DCA, LCA, and UDCA). ^1^Bile acid levels were measured per fresh fecal mass

We used cluster analysis to categorize the participants based on the composition of their fecal BAs. Consequently, two clusters were generated, one with a high level of CA and CDCA (Fig. 3, cluster 4) labeled as high-priBA and the other (Fig. 3, clusters 1–3) predominated by DCA and LCA, and further divided into tertiles based on the total concentration of DCA and LCA, labeled as low-secBA, medium-secBA, and high-secBA, respectively. Consequently, the participants were divided into four clusters (Fig. 3).

### Differences in defecation status among the various BA clusters

Table 2 showed the differences in fecal BA levels and defecation status among BA clusters, where the levels of UDCA differed significantly (ANOVA, *p* = 0.034). The high-priBA cluster had the highest levels (Games–Howell’s test; low-secBA vs. high-priBA, *p* = 0.029; medium-secBA vs. high-priBA, *p* = 0.043; high-secBA vs. high-priBA, *p* = 0.054). On the other hand, the frequency of normal feces differed significantly among the BA clusters (ANOVA, *p* = 0.005), where the low-secBA and high-priBA cluster had significantly more-frequent normal feces than high-secBA (Tukey’s post hoc test, *p* = 0.015; and *p* = 0.011, respectively).

**Table 2:** Fecal bile acid levels and defecation frequency of the study participants living in the community disaggregated by the composition of fecal bile acid

	Cluster 1: low-secBA	Cluster 2: medium-secBA	Cluster 3: high-secBA	Cluster 4: high-priBA	*p*
Total bile acids (µmol/g)^1^	1.50 ± 0.81^a^	2.60 ± 0.66^b^	5.95 ± 2.42^c^	5.36 ± 2.52^c^	< 0.001
CA	0.19 ± 0.27^a^	0.06 ± 0.14^a^	0.09 ± 0.19^a^	2.25 ± 0.71^b^	< 0.001
CDCA	0.10 ± 0.14^a^	0.06 ± 0.11^a^	0.09 ± 0.18^a^	1.28 ± 1.12^b^	0.004
DCA	0.63 ± 0.40^a^	1.42 ± 0.46^b^	3.40 ± 1.54^c^	0.78 ± 0.93^ab^	< 0.001
LCD	0.34 ± 0.31^a^	0.90 ± 0.45^b^	2.09 ± 1.01^c^	0.11 ± 0.13^d^	< 0.001
UDCA	0.10 ± 0.10^a^	0.12 ± 0.25^a^	0.13 ± 0.24^ab^	0.58 ± 0.55^b^	0.034
7-oxo-DCA	0.12 ± 0.26	0.04 ± 0.07	0.03 ± 0.06	0.25 ± 0.31	0.059
BSFS value of the analyzed fecal samples	3.6 ± 1.1	3.3 ± 1.2	3.4 ± 1.3	4.1 ± 1.2	0.222
N	18	18	17	14	
Gender (% females)	77.8 (n = 14)	100	94.1 (n = 16)	100	
BMI (kg/m^2^)	21.5 ± 2.7^ab^	20.0 ± 2.5^a^	20.9 ± 1.8^ab^	22.8 ± 2.8^b^	0.023
Defecation and probiotic status					
Total defecation frequency (time/week)	9.9 ± 5.1^ab^	8.6 ± 3.0^ab^	6.6 ± 2.7^a^	10.4 ± 4.3^b^	0.029
Hard feces (BSFS types 1–2)	1.6 ± 2.8	2.3 ± 3.0	1.6 ± 2.1	0.6 ± 0.9	0.077
Normal feces (BSFS types 3–5)	8.2 ± 4.0^a^	6.1 ± 3.1 ^ab^	4.4 ± 2.8^b^	8.6 ± 4.6^a^	0.005
Watery feces (BSFS types 6–7)	0.2 ± 0.4	0.2 ± 0.4	0.6 ± 0.9	1.2 ± 2.2	0.099
Weekly mean score of BSFS	3.6 ± 0.6^ab^	3.3 ± 1.0 ^a^	3.5 ± 1.1^ab^	4.2 ± 0.6^b^	0.028
Probiotic food intake (time/week)	2.1 ± 2.3	3.3 ± 2.9	2.9 ± 2.3	2.5 ± 1.7	0.491

Data presented as % or mean ± standard deviation. Data are referred to a single sample per participant

*priBA* primary bile acid, *secBA* secondary bile acid, *CA* cholic acid, *CDCA* chenodeoxycholic acid, *DCA* deoxycholic acid, *LCA* Lithocholic acid, *UDCA* ursodeoxycholic acid, *BMI* body mass index, *BSFS* Bristol stool form scale

^1^Bile acid levels were measured per fresh fecal mass

^abcd^Different letters indicate statistically significant differences between the cluster (Tukey’s post hoc test or Games–Howell’s test, *p* < 0.05)

### Differences in intestinal microbiota among BA clusters

Table 3 showed the different intestinal microbiota among BA clusters. The relative abundance of *Bacteroides* in the high-priBA cluster was significantly lower than the low-, medium-, and high-secBA clusters (Tukey’s post hoc test, *p* < 0.001; *p* < 0.001; and *p* = 0.003, respectively). There was significantly less *Clostridium* cluster IV in the high-priBA cluster than the high-secBA cluster (Tukey’s post hoc test, *p* = 0.009). Additionally, there was significantly more *Clostridium* subcluster XIVa in the high-priBA cluster compared to the low-secBA cluster (Games–Howell’s test, *p* = 0.029).

**Table 3:** Composition of the intestinal microbiota of the study participants living in the community disaggregated by the composition of fecal bile acid

	Cluster 1: low-secBA	Cluster 2: medium-secBA	Cluster 3: high-secBA	Cluster 4: high-priBA	*p*
N	18	18	17	14	
*Bifidobacterium* (%)	22.0 ± 11.8	18.1 ± 10.5	18.9 ± 14.5	18.3 ± 13.7	0.784
*Lactobacillales* (Order) (%)	7.3 ± 5.2	7.0 ± 7.4	6.8 ± 5.6	8.7 ± 6.6	0.840
*Bacteroides* (%)	33.2 ± 11.8^a^	34.6 ± 10.9^a^	32.1 ± 12.5^a^	16.9 ± 10.3^b^	< 0.001
*Prevotella* (%)	0.7 ± 2.8	0.0 ± 0.0	0.0 ± 0.0	0.3 ± 0.7	
*Clostridium* cluster IV (%)	7.0 ± 4.2^ab^	7.1 ± 3.7^ab^	8.5 ± 4.3^a^	3.8 ± 3.2^b^	0.015
*Clostridium* subcluster XIVa (%)	18.4 ± 7.6^a^	20.1 ± 10.3^ab^	22.8 ± 7.6^ab^	31.8 ± 14.8^b^	0.030
*Clostridium* cluster IX (%)	3.1 ± 5.0	3.8 ± 4.7	2.3 ± 2.0	6.8 ± 7.0	0.081
*Clostridium* cluster XI (%)	0.7 ± 1.6	0.8 ± 0.8	0.3 ± 0.5	0.3 ± 0.5	0.401
*Clostridium* cluster XVIII (%)	1.2 ± 1.8	0.8 ± 0.8	1.8 ± 3.9	1.2 ± 2.2	0.662
Others (%)	6.5 ± 3.1^a^	7.8 ± 5.6^ab^	6.6 ± 2.6^a^	11.9 ± 6.6^b^	0.006
Firmicutes/Bacteroidetes ratio	1.3 ± 0.5^a^	1.3 ± 0.5^a^	2.3 ± 3.3^ab^	5.5 ± 5.3^b^	0.032

Data presented as mean ± standard deviation. Data are referred to a single sample per participant

*priBA* primary bile acid, *secBA* secondary bile acid

^ab^Different letters indicate statistically significant differences between the cluster (Tukey’s post hoc test or Games–Howell’s test, *p* < 0.05)

### Differences in habitual diet among BA clusters

Table 4 indicates the dietary characteristics of the study participants among BA clusters. The intake of animal protein and animal fat differed significantly among BA clusters (ANOVA, *p* = 0.012 and *p* = 0.002, respectively), with the low-secBA cluster having the lowest intake. However, the intake of insoluble fiber was significantly higher for high-priBA cluster (ANOVA, *p* = 0.022).

**Table 4:** Dietary characteristics of the study participants living in the community disaggregated by the composition of fecal bile acid

	Cluster 1: low-secBA	Cluster 2: medium-secBA	Cluster 3: high-secBA	Cluster 4: high-priBA	*p*
N	18	18	17	14	
Protein (g/1000 kcal)	33.7 ± 4.0	37.6 ± 4.6	37.4 ± 4.2	36.7 ± 6.3	0.058
Animal protein (g/1000 kcal)	17.8 ± 4.0 ^a^	22.6 ± 4.8^b^	22.7 ± 4.1^b^	20.4 ± 6.7^ab^	0.012
Plant protein (g/1000 kcal)	15.9 ± 1.4	15.0 ± 2.6	14.7 ± 1.8	16.4 ± 2.8	0.132
Fat (g/1000 kcal)	27.6 ± 4.3 ^a^	33.0 ± 4.5^b^	31.7 ± 4.5^ab^	29.9 ± 7.2^ab^	0.017
Animal fat (g/1000 kcal)	11.9 ± 3.1 ^a^	16.0 ± 3.3^b^	15.4 ± 2.6^b^	13.5 ± 4.2^ab^	0.002
Plant fat (g/1000 kcal)	15.7 ± 3.3	17.1 ± 4.0	16.3 ± 3.3	16.4 ± 3.6	0.742
Cholesterol (mg/1000 kcal)	184 ± 57	236 ± 63	244 ± 68	210 ± 91	0.053
Total dietary fiber (g/1000 kcal)	6.1 ± 1.2^ab^	6.1 ± 1.5^ab^	5.4 ± 1.0^a^	6.7 ± 1.3^b^	0.038
Soluble dietary fiber (g/1000 kcal)	1.5 ± 0.4	1.6 ± 0.5	1.4 ± 0.3	1.7 ± 0.4	0.185
Insoluble dietary fiber (g/1000 kcal)	4.4 ± 0.9^ab^	4.4 ± 1.0 ^ab^	3.8 ± 0.7^a^	4.8 ± 0.9^b^	0.022
Potassium (mg/1000 kcal)	1164 ± 231	1268 ± 288	1157 ± 183	1250 ± 299	0.454
Calcium (mg/1000 kcal)	216 ± 56	246 ± 56	243 ± 72	243 ± 69	0.466

Data presented as mean ± standard deviation

*priBA* primary bile acid, *secBA* secondary bile acid

^ab^Different letters indicate statistically significant differences between the cluster (Tukey’s post hoc test or Games–Howell’s test, *p* < 0.05)

### Principal component analysis

**Fig. 4:**
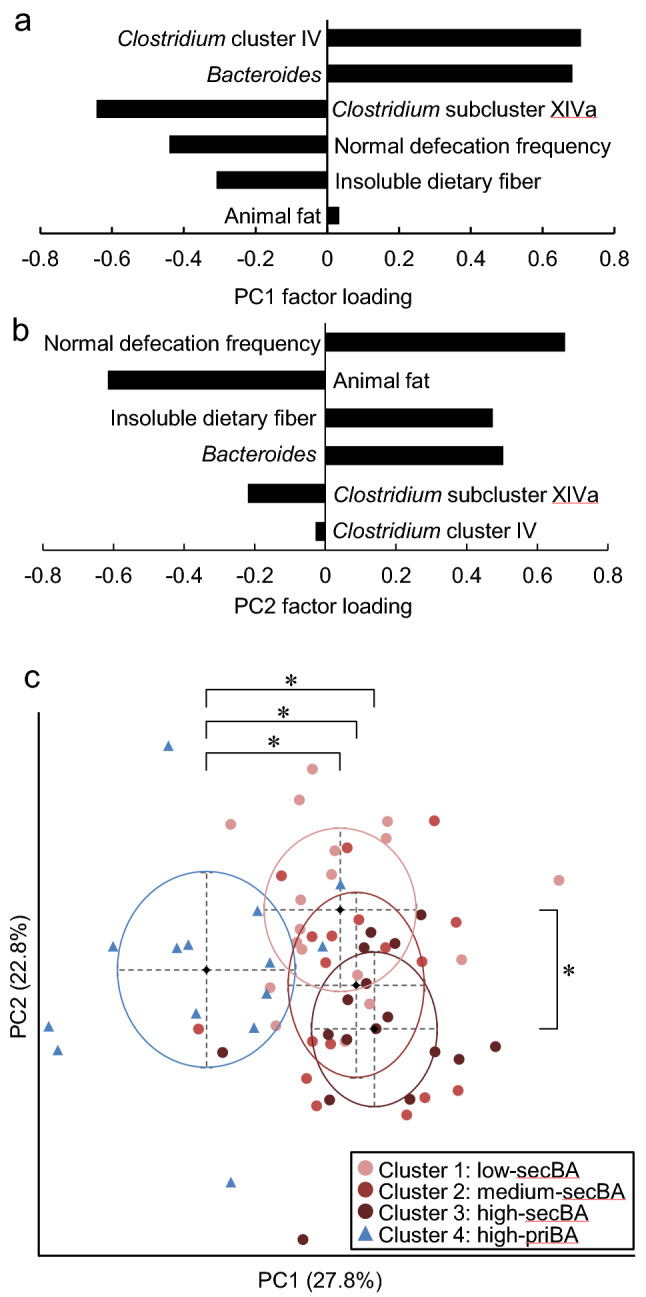
Association of defecation, diet, and intestinal microbiota with fecal bile acid composition in the community-dwelling young participant. Principal component analysis was performed on six variables that showed significant differences among clusters based on the fecal bile acid composition and generated two principal components (PC1 and PC2). **a** Factor loadings of the PC1. **b** Factor loadings of the PC2. **c** PC1 and PC2 plot of the participants according to the bile acid cluster. The center of the ellipse showed the mean values of PC1 and PC2, and the radius showed the standard deviation. PC1 and PC2 were compared among bile acid clusters using a one-way analysis of variance followed by Tukey’s post hoc test. *Tukey’s post hoc test, *p* < 0.001

To better interpret the association of defecation status, intestinal microbiota, and habitual diet with fecal BA composition, we performed a PCA on the six variables that showed significant differences among BA clusters (*Clostridium* cluster IV, *Bacteroides*, *Clostridium* subcluster XIVa, normal defecation frequency, insoluble fiber, and animal fat). Animal protein was excluded because its intake was assumed to be associated with the intake of animal fat. The PCA showed that the first principal component (PC1) explained 27.8% of the variance, whereas the second principal component (PC2) explained 22.8%. The major variables of the PC1 were *Clostridium* cluster IV, *Bacteroides*, and *Clostridium* subcluster XIVa; thus, PC1 was considered as a component of the intestinal microbiota (Fig. 4a). Conversely, the major variables of the PC2 were normal defecation frequency, animal fat, and insoluble fiber; hence, PC2 was considered as a component of the defecation and dietary status. *Bacteroides* was a major component of the PC1, but was also moderately involved in the PC2 (Fig. 4b).

The distributions of PC1 and PC2 of the participants were shown in Fig. 4c according to BA clusters. The high-priBA cluster in PC1 was significantly lower than the other three clusters (ANOVA, *p* < 0.001, Tukey’s post hoc test; high-priBA vs. low-secBA, *p* < 0.001; high-priBA vs. medium-secBA, *p* < 0.001; high-priBA vs. high-secBA, *p* < 0.001). Conversely, the low-secBA cluster in PC2 was significantly higher than the high-secBA cluster and no significantly higher than the medium-secBA cluster (ANOVA, *p* = 0.002, Tukey’s post hoc test, low-priBA vs. high-secBA, *p* < 0.001; low-secBA vs. medium-secBA, *p* = 0.055).

## Discussion

To explore the factors affecting the fecal BA composition, the present study investigated a wide range of variables including defecation status, habitual diet, intestinal microbiota, and fecal BA levels. The participants of the study were classified according to their fecal BA composition. In this study, 20.9% of the participants had high fecal BA levels with predominantly priBAs (CA and CDCA) (high-priBA cluster). This cluster was associated with an increased relative abundance of *Clostridium* subcluster XIVa, increased frequency of normal feces, and decreased relative abundance of *Bacteroides* and *Clostridium* cluster IV. Alternatively, high-secBA cluster, which had the same level of total fecal BAs as high-priBA cluster, but with a predominance of secBAs, was associated with an increased animal fat intake, and decreased frequency of normal feces and insoluble fiber intake.

### Factors related to high fecal priBA levels

Members of *Clostridium* subcluster XIVa may assist with production of secBAs. The *Clostridium* subcluster XIVa contained strains with high BA 7α-dehydroxylating activity [42]. Kakiyama et al. investigated fecal BAs and intestinal microbiota in patients with cirrhosis and healthy participants; they concluded that the relative abundances of *Ruminococcaceae* and *Blautia*—members of *Clostridium* subcluster XIVa—were positively correlated with fecal secBAs levels [43]. Murakami et al. investigated fecal BAs and intestinal microbiota in patients with gastrointestinal diseases and healthy participants and found that the relative abundance of *Clostridium* subcluster XIVa was positively correlated with the 7α-dehydroxylation marker, the DCA/(DCA + CA) ratio [20].

We found the relative abundance of *Clostridium* subcluster XIVa to increase in a near-linear fashion among the various secBA level tertiles, although it was highest in high-priBA. A recent study estimated that BA 7α-dehydroxylation comprised only ~ 0.0001% of the total intestinal microbiota and that most *Clostridium* subcluster XIVa members lacked the bai operon—the gene cluster for the 7α-dehydroxylation [44]. A study that used rats showed that CA feeding increased levels in *Clostridium* subcluster XIVa members [45]. The proliferation of this cluster member may be further promoted by an increased input of CA into the large intestine [46]. On the other hand, the *Clostridium* subcluster XIVa contained butyrate-producing bacteria [47]. Increases in the abundance of *Clostridium* subcluster XIVa in the colons of piglets and mice were observed following dietary fiber supplementation [48, 49].

Zhao et al. examined the BA-related metabolism and metagenome in 290 patients with diarrhea-predominant IBS and 89 healthy participants. Their results indicated UDCA and 7-oxo-DCA may enhance hepatic synthesis and fecal excretion of BAs by attenuation of farnesoid X receptor/fibroblast growth factor 19 signaling [50]. A randomized controlled study in patients with morbid obesity showed that UDCA administration stimulated BA synthesis by reducing circulating fibroblast growth factor 19 and farnesoid X receptor activation [51]. Interestingly, the fecal UDCA and 7-oxo-DCA levels in the high-priBA cluster were significantly higher than levels observed in other clusters. While we don’t know what caused the high fecal priBA levels in the high-priBA cluster, high fiber intake and frequent defecation might have contributed to this finding; additional studies are needed to examine this question. On the other hand, elevated fecal priBA levels were previously observed in individuals with functional bowel disorders and cirrhosis [43, 52].

### Factors involved in high fecal secBA levels

We found high fecal secBA levels to be associated with fewer “normal” bowel movements and a higher intake of animal fat. A randomized controlled-feeding trial of healthy young adults showed that consuming a high-fat diet for 6 months increased fecal secBA levels [53]. A short-term dietary intervention study showed that an animal-based diet significantly increased fecal DCA levels and microbial bile salt hydrolases gene expression [24]. Although several studies showed that high-volume intake of fat or animal foods increased BA secretion and was associated with increased fecal secBA levels [54], these studies did not examine defecation status. Conversely, Thomas et al. investigated the relationship between large bowel transit time and the fecal activity of BA metabolizing enzymes in patients with acromegaly [55] and cholesterol cholelithiasis [56]. They found that prolonged colonic transit time was associated with increased activity of the 7α-dehydroxylating enzyme. Hence, the combination of infrequent bowel movements and intake of a high-fat diet may predispose to increased fecal secBA levels. In the present study, the fat intake in the high-secBA cluster was comparable with that of medium-secBA, although participants in the high-secBA cluster tended to have fewer bowel movements.

High-secBA cluster members, who presumably produced more secBAs, were at increased risk of colon cancer [9], cholelithiasis [10], and liver cancer [11]. Conversely, the high-priBA cluster had lower secBAs, but higher priBAs, as well as more-frequent bowel movements and higher dietary fiber intake. Insoluble fibers can hold large amounts of water; increased intake of insoluble fiber promotes intestinal peristalsis and increases fecal volume [57]. Additionally, fibers can bind BAs [58]; thus, increased consumption of dietary insoluble fiber will promote fecal BA excretion, potentially lowering serum cholesterol [59]. Nevertheless, several studies in patients with IBS showed that high fecal priBA levels might be associated with inflammation and poor prognosis [1].

Our study had some limitations. First, its cohort was relatively small and consisted mostly of female. Fecal BA levels were not significantly different between males and females (data not shown), but sex differences in colorectal motility and the prevalence of functional constipation have been reported [60, 61]. Whether fecal bile acid concentrations vary by sex is an interesting question. The female-only dataset analysis also produced results similar to the analysis that included both males and females (Online Resources 5). Second, the T-RFLP method cannot reveal the role of bacterial species and is inferior to next-generation sequencing. Third, BDHQ can assess false intakes through reporting biases. Fourth, because the BSFS scores were self-reported, we cannot exclude the possibility of recall or other individual-level bias. Finally, this was a cross-sectional study, without a control group; hence, causality could not be determined.

## Conclusion

High fecal priBA levels were associated with a low relative abundance of *Clostridium* cluster IV and *Bacteroides*, a high relative abundance of *Clostridium* subcluster XIVa, and a high normal defecation frequency. Although the health effects of high fecal priBA or each *Clostridium* cluster remain unclear, our results provide important insights for regulation of intestinal BA metabolism. Conversely, high levels of cytotoxic secBA were associated with low normal defecation frequency, low insoluble fiber intake, and high animal fat intake. These results indicate that among community-dwelling young adults, secBA production is affected by both dietary and lifestyle-related factors. These results may inform novel strategies for preventing colorectal cancer and cholelithiasis.

## Supplementary Information

Below is the link to the electronic supplementary material.

Supplementary file1 (XLSX 17 KB)

Supplementary file2 (PDF 32 KB)

Supplementary file3 (XLSX 17 KB)

Supplementary file4 (PDF 131 KB)

Supplementary file5 (PDF 273 KB)

## Data Availability

Deidentified data are available from the corresponding author upon reasonable request.
