# Supplementary material for: Defecation status, intestinal microbiota, and habitual diet are associated with the fecal bile acid composition: a cross-sectional study in community-dwelling young participants
Source: Eur J Nutr. 2023 Mar 7;62(5):2015–26. doi: 10.1007/s00394-023-03126-8 (PMC10349756; doi:10.1007/s00394-023-03126-8)

Online Resource 2

Article title: Defecation status, intestinal microbiota, and habitual diet are associated with the fecal bile acid composition: A cross-sectional study in community-dwelling young participants
Journal name: *European Journal of Nutrition*
Author names: Yosuke Saito* and Toyoaki Sagae
***Corresponding author:** Department of Clinical Nutrition, Faculty of Health and Wellness Sciences, Hiroshima International University, E-mail: saito-y@hirokoku-u.ac.jp

Figure Captions

Fig. S1 Comparison of bile acid level profiles between categorizations obtained by cluster analysis that did not include BSFS type (a) and those obtained by cluster analysis that did (b). Abbreviations: DCA, deoxycholic acid; LCA, lithocholic acid; CA, cholic acid; CDCA, chenodeoxycholic acid; UDCA, ursodeoxycholic acid; G-BA, glycine conjugated bile acid; T-BA, taurine conjugated bile acid; priBA, primary bile acid; secBA, secondary bile acid. G-BA and T-BA each contained five bile acids (CA, CDCA, DCA, LCA, and UDCA). [1] Bile acid levels were measured per fresh fecal mass

Fig. S1

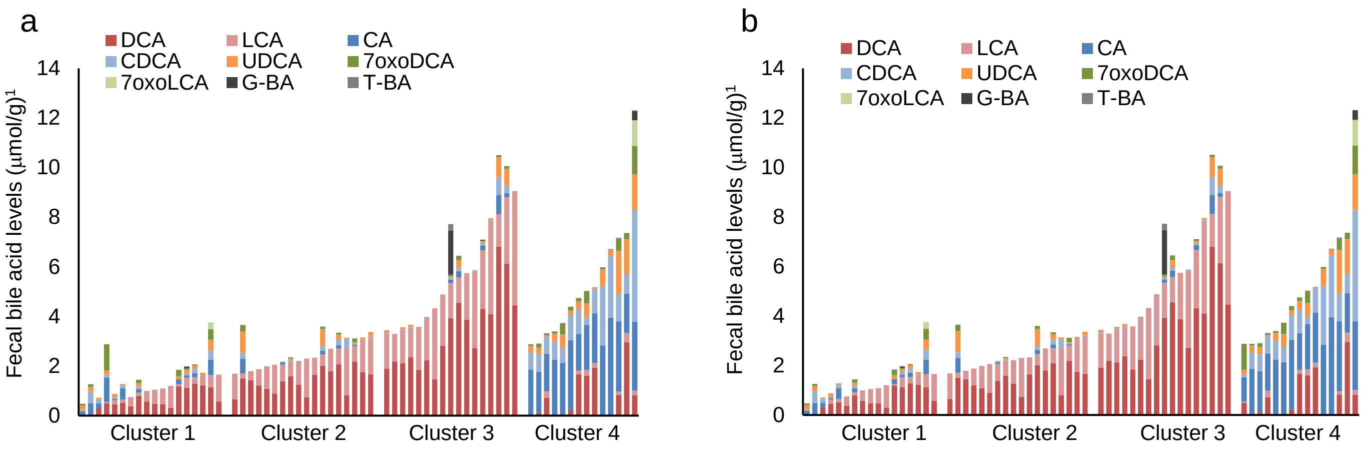

Supplement: Supplementary file 2 — Supplementary file2 (PDF 32 KB) [file 394_2023_3126_MOESM2_ESM.pdf]
