# Supplementary material for: Defecation status, intestinal microbiota, and habitual diet are associated with the fecal bile acid composition: a cross-sectional study in community-dwelling young participants
Source: Eur J Nutr. 2023 Mar 7;62(5):2015–26. doi: 10.1007/s00394-023-03126-8 (PMC10349756; doi:10.1007/s00394-023-03126-8)
Supplement: Supplementary file 4 — Supplementary file4 (PDF 131 KB) [file 394_2023_3126_MOESM4_ESM.pdf]

Online Resource 4

Article title: Defecation status, intestinal microbiota, and habitual diet are associated with the fecal bile acid composition: A cross-sectional study in community-dwelling young participants

Journal name: *European Journal of Nutrition*

Author names: Yosuke Saito* and Toyoaki Sagae
***Corresponding author:** Department of Clinical Nutrition, Faculty of Health and Wellness Sciences, Hiroshima International University, E-mail: saito-y@hirokoku-u.ac.jp

Table S1. Characteristics of study young participants living in the community

	All participants (n = 67)
Age (year)	19.9 ± 0.9
Gender (% females)	92.5 (n = 62)
Height (cm)	158 ± 6
Body weight (kg)	52.8 ± 7.4
BMI (kg/m^2)	21.2 ± 2.6
Habitual diet	
Protein (g/1000 kcal)	36.3 ± 4.9
Animal protein (g/1000 kcal)	20.8 ± 5.2
Plant protein (g/1000 kcal)	15.5 ± 2.2
Fat (g/1000 kcal)	30.6 ± 5.4
Animal fat (g/1000 kcal)	14.2 ± 3.6
Plant fat (g/1000 kcal)	16.4 ± 3.5
Cholesterol (mg/1000 kcal)	219 ± 72
Total dietary fiber (g/1000 kcal)	6.1 ± 1.3
Soluble dietary fiber (g/1000 kcal)	1.6 ± 0.4
Insoluble dietary fiber (g/1000 kcal)	4.4 ± 0.9
Potassium (mg/1000 kcal)	1208 ± 252
Calcium (mg/1000 kcal)	236 ± 63

Data presented as % or mean ± standard deviation.
Abbreviations: BMI, body mass index
