# Supplementary material for: Defecation status, intestinal microbiota, and habitual diet are associated with the fecal bile acid composition: a cross-sectional study in community-dwelling young participants
Source: Eur J Nutr. 2023 Mar 7;62(5):2015–26. doi: 10.1007/s00394-023-03126-8 (PMC10349756; doi:10.1007/s00394-023-03126-8)

Online Resource 5

Article title: Defecation status, intestinal microbiota, and habitual diet are associated with the fecal bile acid composition: A cross-sectional study in community-dwelling young participants
Journal name: *European Journal of Nutrition*
Author names: Yosuke Saito* and Toyoaki Sagae
***Corresponding author:** Department of Clinical Nutrition, Faculty of Health and Wellness Sciences, Hiroshima International University, E-mail: saito-y@hirokoku-u.ac.jp

Table S2. Characteristics of only female participants in this study

	**All participants (n = 62)**
Age (year)	19.9 ± 0.9
Gender (% females)	100
Height (cm)	156.8 ± 5.4
Body weight (kg)	52.5 ± 7.5
BMI (kg/m^2)	21.3 ± 2.7
Habitual diet	
Protein (g/1000 kcal)	36.5 ± 5.0
Animal protein (g/1000 kcal)	21.1 ± 5.3
Plant protein (g/1000 kcal)	15.5 ± 2.3
Fat (g/1000 kcal)	31.1 ± 5.3
Animal fat (g/1000 kcal)	14.4 ± 3.7
Plant fat (g/1000 kcal)	16.7 ± 3.4
Cholesterol (mg/1000 kcal)	220 ± 72
Total dietary fiber (g/1000 kcal)	6.1 ± 1.4
Soluble dietary fiber (g/1000 kcal)	1.6 ± 0.4
Insoluble dietary fiber (g/1000 kcal)	4.4 ± 0.9
Potassium (mg/1000 kcal)	1225 ± 251
Calcium (mg/1000 kcal)	240 ± 63

Data presented as % or mean ± standard deviation.
Abbreviations: BMI, body mass index

Table S3. Bile acid levels and microbiota in the feces of only female participants in this study

	**All participants (n = 62)**
Bile acid levels (µmol/g) [1]	
Total bile acids	3.86 ± 2.54
CA	0.59 ± 0.98
CDCA	0.36 ± 0.73
DCA	1.63 ± 1.48
LCD	0.90 ± 0.98
UDCA	0.22 ± 0.37
7-oxo-DCA	0.10 ± 0.22
7-oxo-LCA	0.02 ± 0.13
Glycine conjugated bile acid [2]	0.04 ± 0.23
Taurine conjugated bile acid [2]	0.01 ± 0.03
Microbiota (%)	
Bifidobacterium	19.1 ± 12.7
Lactobacillales (Order)	7.6 ± 6.3
Bacteroides	30.0 ± 13.3
Prevotella	0.2 ± 1.5
Clostridium cluster IV	6.6 ± 4.3
Clostridium subcluster XIVa	22.8 ± 11.5
Clostridium cluster IX	4.0 ± 5.2
Clostridium cluster XI	0.6 ± 1.0
Clostridium cluster XVIII	1.1 ± 2.3
Others	8.0 ± 5.2
Firmicutes/Bacteroidetes ratio	2.5 ± 3.5

Data presented as mean ± standard deviation. Data are referred to a single sample per participant. Abbreviations: CA, cholic acid; CDCA, chenodeoxycholic acid; DCA, deoxycholic acid; LCA, lithocholic acid; UDCA, ursodeoxycholic acid.

[1] Bile acid levels were measured per fresh fecal mass

[2] Glycine and taurine conjugated bile acid each contained five bile acids (CA, CDCA, DCA, LCA, and UDCA)

Table S4. Fecal bile acid levels and defecation frequency of only female participants in this study disaggregated by the composition of fecal bile acid

	**Cluster 1: low-secBA**	**Cluster 2: medium-secBA**	**Cluster 3: high-secBA**	**Cluster 4: high-priBA**	***p***
Total bile acids (μmol/g) [1]	1.59 ± 0.81 [a]	2.59 ± 0.60 [b]	6.10 ± 2.42 [c]	5.36 ± 2.52 [c]	<0.001
CA	0.19 ± 0.27 [a]	0.03 ± 0.05 [a]	0.09 ± 0.20 [a]	2.25 ± 0.71 [b]	<0.001
CDCA	0.10 ± 0.13 [a]	0.06 ± 0.10 [a]	0.10 ± 0.18 [a]	1.28 ± 1.12 [b]	0.004
DCA	0.68 ± 0.45 [a]	1.47 ± 0.45 [b]	3.49 ± 1.54 [c]	0.78 ± 0.93 [ab]	<0.001
LCD	0.36 ± 0.37 [a]	0.93 ± 0.45 [b]	2.11 ± 1.04 [c]	0.11 ± 0.13 [a]	<0.001
UDCA	0.14 ± 0.20 [ab]	0.08 ± 0.17 [a]	0.14 ± 0.25 [ab]	0.58 ± 0.55 [b]	0.029
7-oxo-DCA	0.12 ± 0.26	0.03 ± 0.05	0.03 ± 0.06	0.25 ± 0.31	0.054
BSFS value of the analyzed fecal samples	3.3 ± 1.1	3.4 ± 1.3	3.3 ± 1.3	4.1 ± 1.2	0.191
N	16	16	16	14	
Gender (% females)	100	100	100	100	
BMI (kg/m^2)	22.0 ± 2.8 [ab]	19.9 ± 2.4 [a]	21.0 ± 1.9 [ab]	22.8 ± 2.8 [b]	0.015
Defecation and probiotic status					
Total defecation frequency (time/week)	8.9 ± 5.0	8.8 ± 3.1	6.6 ± 2.8	10.4 ± 4.3	0.065
Hard feces (BSFS types 1–2)	1.8 ± 2.9	2.6 ± 3.1	1.7 ± 2.2	0.6 ± 0.9	0.189
Normal feces (BSFS types 3–5)	6.9 ± 3.0 [ab]	5.9 ± 3.3 [ab]	4.2 ± 2.8 [a]	8.6 ± 4.6 [b]	0.009
Watery feces (BSFS types 6–7)	0.2 ± 0.4	0.2 ± 0.4	0.7 ± 0.9	1.2 ± 2.2	0.110
Weekly mean score of BSFS	3.5 ± 0.6 [a]	3.3 ± 1.1 [ab]	3.4 ± 1.1 [ab]	4.2 ± 0.6 [b]	0.019
Probiotic food intake (time/week)	2.6 ± 2.5	3.2 ± 2.9	3.0 ± 2.4	2.5 ± 1.7	0.833

Data presented as % or mean ± standard deviation. Data are referred to a single sample per participant. Abbreviations: priBA, primary bile acid; secBA, secondary bile acid; CA, cholic acid; CDCA, chenodeoxycholic acid; DCA, deoxycholic acid; LCA, Lithocholic acid; UDCA, ursodeoxycholic acid; BMI, body mass index; BSFS, Bristol stool form scale

[1] Bile acid levels were measured per fresh fecal mass

[abc] Different letters indicate statistically significant differences between the cluster (Tukey's post hoc test or Games–Howell's test, $p < 0.05$)

Table S5. Composition of the intestinal microbiota of only female participants living in the community disaggregated by the composition of fecal bile acid

	**Cluster 1: low-secBA**	**Cluster 2: medium-secBA**	**Cluster 3: high-secBA**	**Cluster 4: high-priBA**	***p***
N	16	16	16	14	
Bifidobacterium (%)	21.6 ± 12.0	17.3 ± 10.6	19.1 ± 15.0	18.3 ± 13.7	0.803
Lactobacillales (Order) (%)	8.7 ± 6.7	6.1 ± 6.6	7.0 ± 5.7	8.7 ± 6.6	0.602
Bacteroides (%)	35.0 ± 11.2 [a]	35.1 ± 11.5 [a]	31.2 ± 12.5 [a]	16.9 ± 10.3 [b]	<0.001
Prevotella (%)	0.7 ± 3.0	0.0 ± 0.0	0.0 ± 0.0	0.3 ± 0.7	
Clostridium cluster IV (%)	6.6 ± 4.5 [ab]	7.3 ± 3.8 [ab]	8.4 ± 4.4 [a]	3.8 ± 3.2 [b]	0.023
Clostridium subcluster XIVa (%)	16.0 ± 7.3 [a]	21.3 ± 10.3 [ab]	23.2 ± 7.6 [ab]	31.8 ± 14.8 [b]	0.005
Clostridium cluster IX (%)	3.1 ± 5.3	4.2 ± 4.9	2.1 ± 2.0	6.8 ± 7.0	0.089
Clostridium cluster XI (%)	0.8 ± 1.7	0.8 ± 0.8	0.4 ± 0.5	0.3 ± 0.5	0.406
Clostridium cluster XVIII (%)	0.7 ± 0.7	0.8 ± 0.8	1.9 ± 4.0	1.2 ± 2.2	0.474
Others (%)	6.6 ± 4.0 [a]	7.2 ± 5.4 [a]	6.7 ± 2.6 [a]	11.9 ± 6.6 [b]	0.011
Firmicutes/Bacteroidetes ratio	1.1 ± 0.4 [a]	1.3 ± 0.5 [a]	2.4 ± 3.4 [ab]	5.5 ± 5.3 [b]	0.018

Data presented as mean ± standard deviation. Data are referred to a single sample per participant. Abbreviations: priBA, primary bile acid; secBA, secondary bile acid

[ab] Different letters indicate statistically significant differences between the cluster (Tukey's post hoc test or Games–Howell's test, $p < 0.05$)

Table S6. Dietary characteristics of only female participants living in the community disaggregated by the composition of fecal bile acid

	**Cluster 1: low-secBA**	**Cluster 2: medium-secBA**	**Cluster 3: high-secBA**	**Cluster 4: high-priBA**	***p***
N	16	16	16	14	
Protein (g/1000 kcal)	34.4 ± 4.3	37.5 ± 4.8	37.5 ± 4.3	36.7 ± 6.3	0.232
Animal protein (g/1000 kcal)	18.1 ± 4.2 [a]	22.8 ± 5.0 [b]	22.9 ± 4.1 [b]	20.4 ± 6.7 [ab]	0.027
Plant protein (g/1000 kcal)	16.3 ± 1.7	14.7 ± 2.4	14.7 ± 1.8	16.4 ± 2.8	0.043
Fat (g/1000 kcal)	28.9 ± 3.8 [a]	33.3 ± 4.7 [b]	32.0 ± 4.4 [ab]	29.9 ± 7.2 [ab]	0.040
Animal fat (g/1000 kcal)	12.2 ± 3.2 [a]	16.2 ± 3.4 [b]	15.5 ± 2.6 [b]	13.5 ± 4.2 [ab]	0.005
Plant fat (g/1000 kcal)	16.8 ± 2.5	17.1 ± 4.2	16.5 ± 3.2	16.4 ± 3.6	0.956
Cholesterol (mg/1000 kcal)	195 ± 62	235 ± 67	238 ± 64	210 ± 91	0.284
Total dietary fiber (g/1000 kcal)	6.4 ± 1.1 [ab]	6.0 ± 1.6 [ab]	5.3 ± 1.0 [a]	6.7 ± 1.3 [b]	0.025
Soluble dietary fiber (g/1000 kcal)	1.6 ± 0.3	1.6 ± 0.5	1.4 ± 0.4	1.7 ± 0.4	0.164
Insoluble dietary fiber (g/1000 kcal)	4.7 ± 0.8 [a]	4.3 ± 1.1 [ab]	3.8 ± 0.7 [b]	4.8 ± 0.9 [a]	0.010
Potassium (mg/1000 kcal)	1244 ± 212	1254 ± 303	1155 ± 189	1250 ± 299	0.650
Calcium (mg/1000 kcal)	233 ± 57	243 ± 58	242 ± 74	243 ± 69	0.964

Data presented as mean ± standard deviation. Abbreviations: priBA, primary bile acid; secBA, secondary bile acid

[ab] Different letters indicate statistically significant differences between the cluster (Tukey’s post hoc test or Games–Howell's test, $p < 0.05$)

Figure Captions

Fig. S2 Defecation status for a week in only female participants. The participants assessed and recorded all feces excreted in the week using the BSFS. (a) Distribution of weekly mean score of BSFS for each participant. (b) Distribution of frequency of excretion of normal feces (BSFS type 3–5). (c) Distribution of frequency of excretion of hard feces (BSFS type 1–2). (d) Distribution of frequency of excretion of watery feces (BSFS type 6–7). Abbreviations: BSFS, Bristol stool form scale

Fig. S3 Distribution of total bile acid levels according to the form of the analyzed feces in only female participants. The form of the feces used in the analysis was assessed and recorded by the participants using the BSFS at the time of fecal collection. BSFS types 1 and 2 indicate hard stools, 3-5 indicate normal stools, and 6 and 7 indicate watery stools. Abbreviations: BSFS, Bristol stool form scale. 1 Bile acid levels were measured per fresh fecal mass

Fig. S2

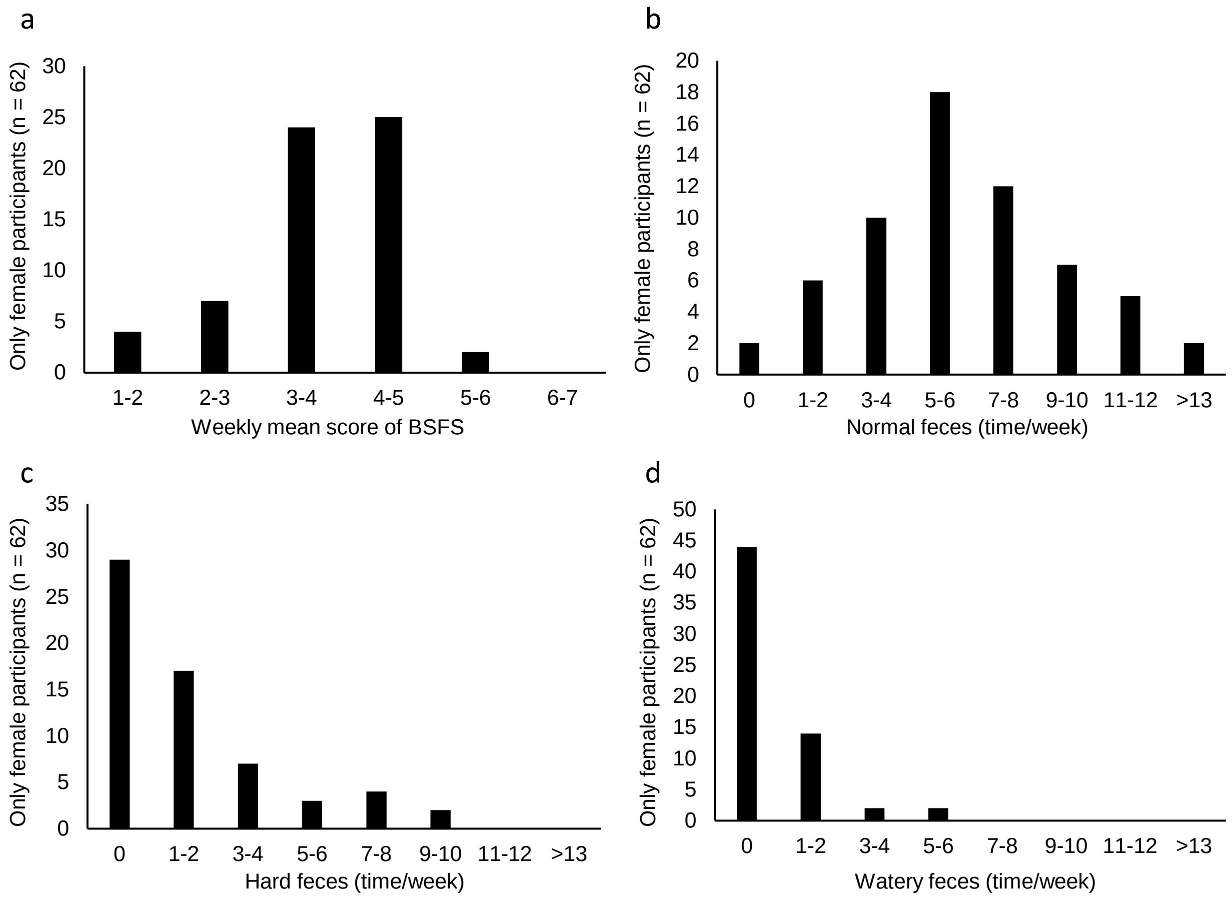

Fig. S3

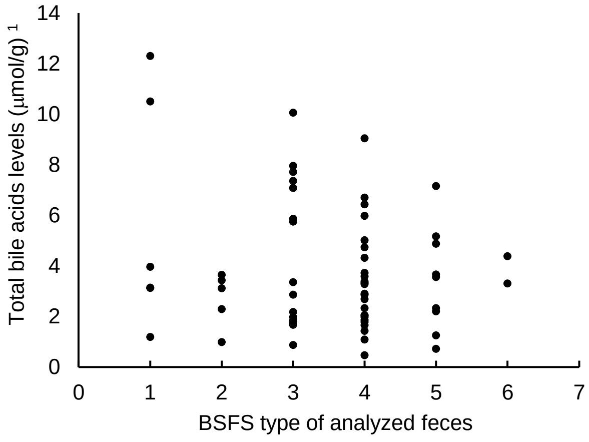

Figure Captions

Fig. S4 Profiling of fecal bile acids of the only female participants. Abbreviations: DCA, deoxycholic acid; LCA, lithocholic acid; CA, cholic acid; CDCA, chenodeoxycholic acid; UDCA, ursodeoxycholic acid; G-BA, glycine conjugated bile acid; T-BA, taurine conjugated bile acid; priBA, primary bile acid; secBA, secondary bile acid. G-BA and T-BA each contained five bile acids (CA, CDCA, DCA, LCA, and UDCA). 1 Bile acid levels were measured per fresh fecal mass

Fig. S5 Association of defecation, diet, and intestinal microbiota with fecal bile acid composition in the only female participants. Principal component analysis was performed on six variables that showed significant differences among clusters based on the fecal bile acid composition and generated two principal components (PC1 and PC2). (a) Factor loadings of the PC1. (b) Factor loadings of the PC2. (c) PC1 and PC2 plot of the participants according to the bile acid cluster. The center of the ellipse showed the mean values of PC1 and PC2, and the radius showed the standard deviation. PC1 and PC2 were compared among bile acid clusters using a one-way analysis of variance followed by Tukey's post hoc test. * Tukey's post hoc test, $p < 0.001$

Fig. S4

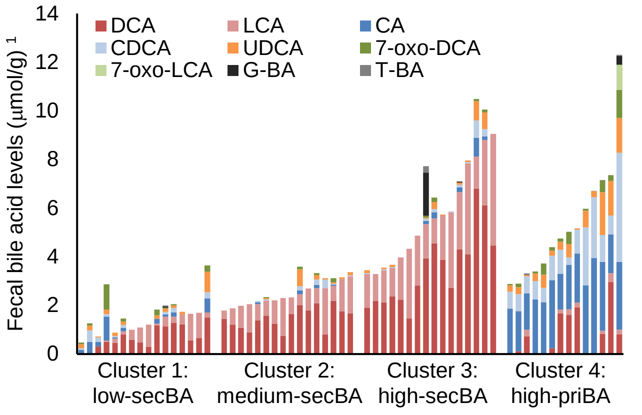

Fig. S5

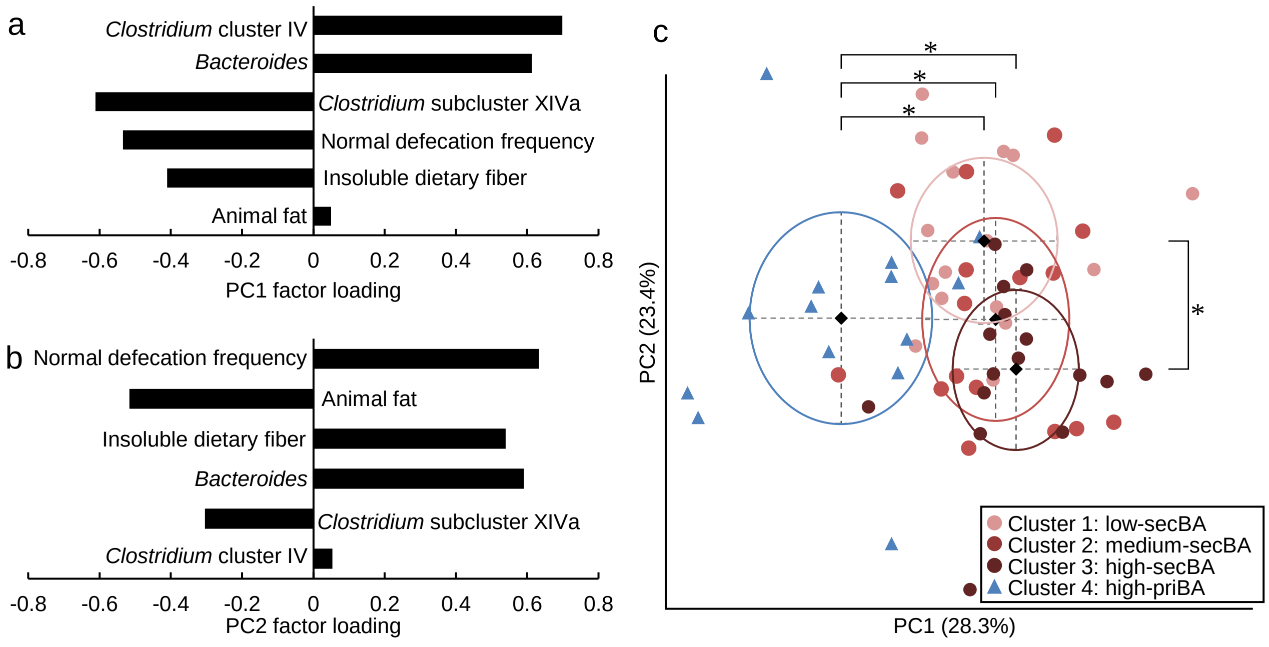

Supplement: Supplementary file 5 — Supplementary file5 (PDF 273 KB) [file 394_2023_3126_MOESM5_ESM.pdf]
